# Supplementary material for: Estimating the Reduction in Genetic Diversity from Background Selection under Non-equilibrium Demography and Partial Selfing
Source: Mol Biol Evol. 2026 Jan 7;43(2):msag004. doi: 10.1093/molbev/msag004 (PMC12902155; doi:10.1093/molbev/msag004)
Supplement: msag004_Supplementary_Data [file msag004_supplementary_data.pdf]

786 # Supplementary Materials

787 ## Supplementary Figures

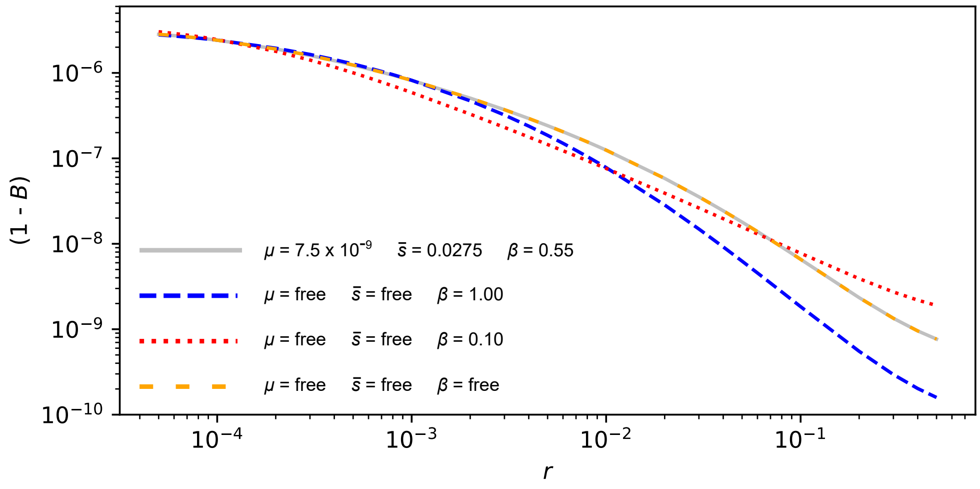

Figure S1: Analytic predictions from classic-BGS theory for the reduction in genetic diversity due to BGS. The predicted departure from neutral levels of diversity ($1 - B$, y-axis) is plotted against the recombination distance of a selected site (r, x-axis). The grey line shows predictions for a set of DFE parameters ($\mu = 7.5 \times 10^{-9}$, $\bar{s} = 0.0275$, $\beta = 0.55$). The dashed blue line is the prediction achieved from minimising the mean squared error to the original predictions (grey line) while enforcing $\beta = 1.00$, but allowing the other parameters to vary freely. The dotted red line shows the same for $\beta = 0.10$, whereas the dashed orange line is the prediction achieved when allowing all three parameters to vary freely. The poor match between predictions when β is misspecified suggests that this parameter is identifiable under this model.

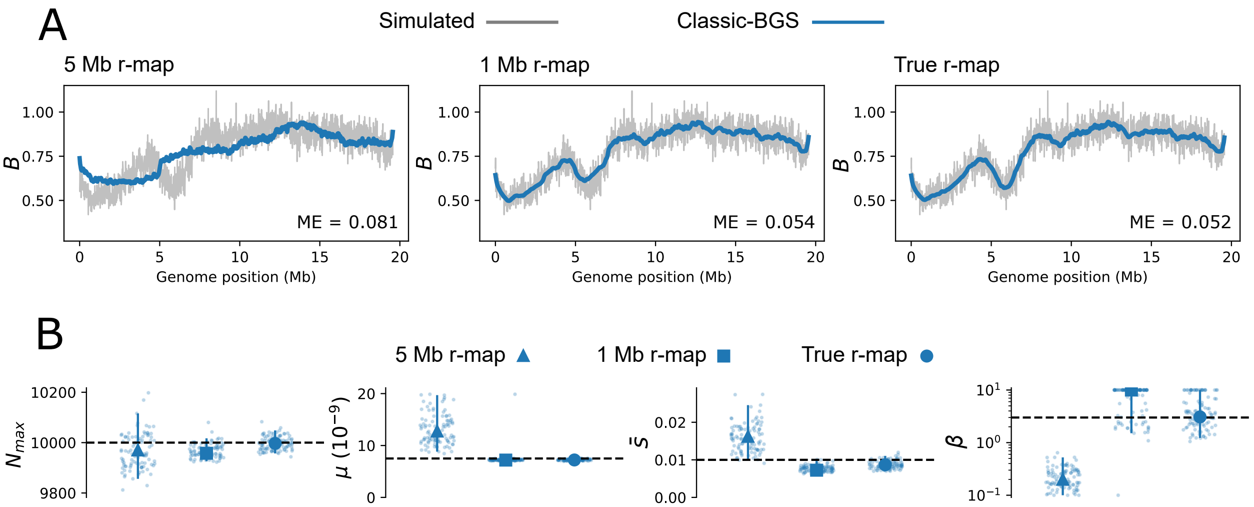

Figure S2: B-maps and parameter estimates from the classic-BGS model under varying recombination map resolution. **(A):** The simulated reduction in nucleotide diversity (B) generated by BGS along one simulated chromosome is shown in grey, with the estimated B-map from the classic-BGS model shown as a blue line. The mean error (ME) between the simulated and predicted B-map is shown in the bottom-right corner of each panel. The panels vary in the resolution of the recombination map that was used for model fitting. **(B):** Estimates of N_{max}, μ, $\bar{s}$ and β from the classic-BGS model. Point estimates are shown as large points, with shape corresponding to the resolution of the recombination map. Bootstrap estimates are shown as small jittered points and 95% CLs are shown as vertical lines. Dashed horizontal lines in each panel correspond to the simulated parameter values.

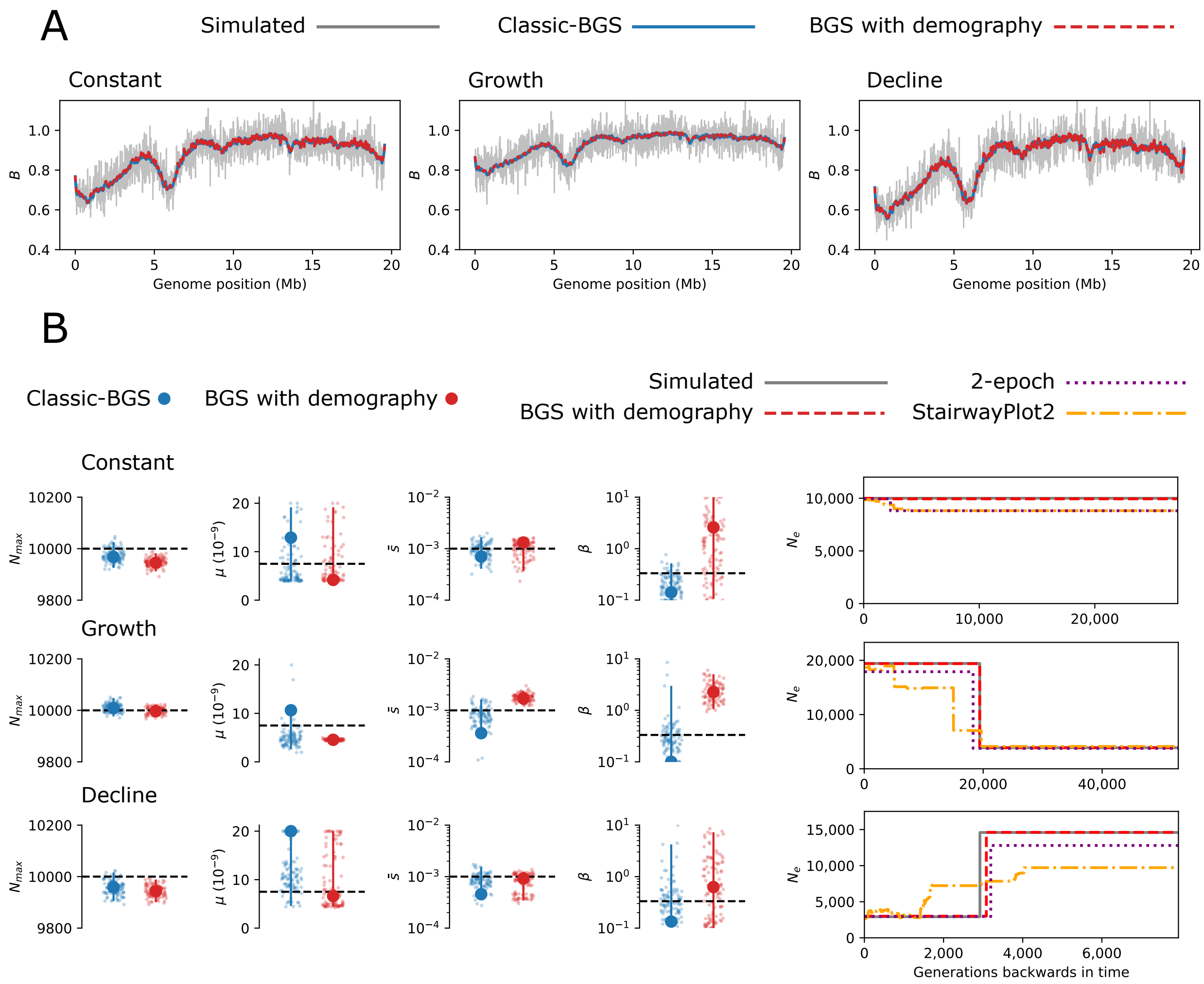

Figure S3: Estimation of BGS under non-equilibrium demography and weak selection. **(A):** The reduction in genetic diversity from BGS along one chromosome is shown for simulations with constant population size, population growth and population decline. The grey line in each panel is the observed reduction in the simulation, whereas estimates from the classic-BGS and BGS-with-demography models are shown as blue and dashed-red lines, respectively. **(B)**: Parameter estimates from the classic-BGS and BGS-with-demography model under each demographic scenario (see Figure S2). The demographic history of each simulation scenario is shown on the right. The grey line corresponds to the number of individuals (N) of the simulated Wright-Fisher population. Estimates from the BGS-with-demography model are shown as a dashed-red line and the estimated history from methods assuming selective neutrality are also plotted for comparison.

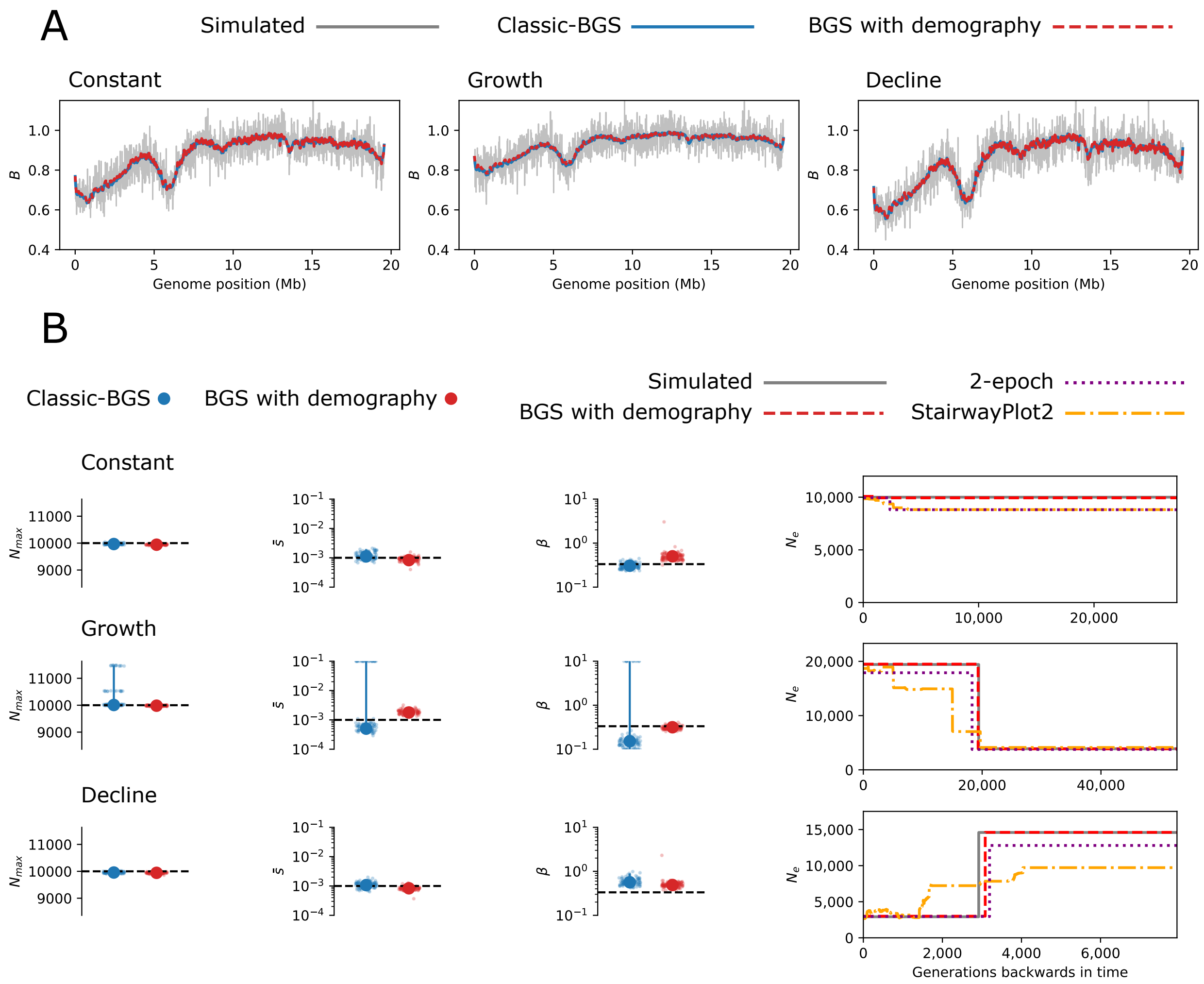

Figure S4: Estimation of BGS under non-equilibrium demography and weak selection when μ is known. **(A):** The reduction in genetic diversity from BGS along one chromosome is shown for simulations with constant population size, population growth and population decline. The grey line in each panel is the observed reduction in the simulation, whereas estimates from the classic-BGS and BGS-with-demography models are shown as blue and dashed-red lines, respectively. **(B)**: Parameter estimates from the classic-BGS and BGS-with-demography model under each demographic scenario. The demographic history of each simulation scenario is shown on the right. The grey line corresponds to the number of individuals (N) of the simulated Wright-Fisher population. Estimates from the BGS-with-demography model are shown as a dashed-red line and the estimated history from methods assuming selective neutrality are also plotted for comparison.

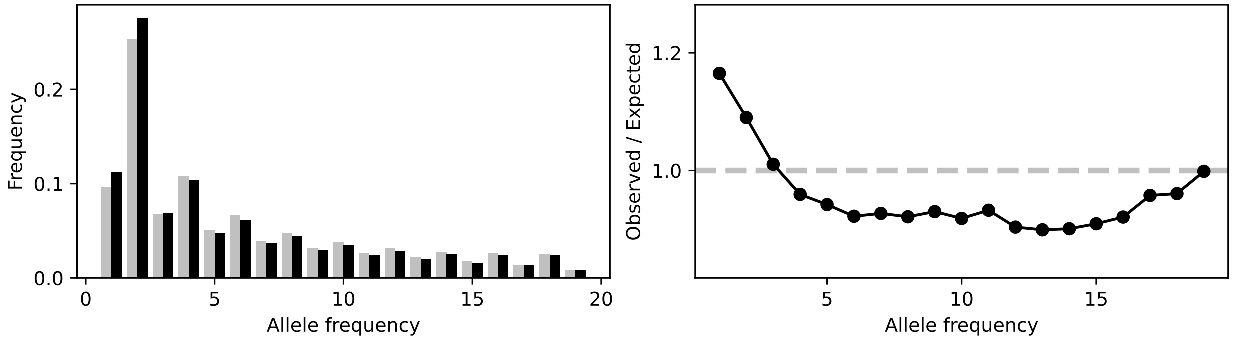

Figure S5: The normalised site frequency spectrum (SFS) for a highly selfing population ($\alpha = 0.9$). The left plot shows spectra as bar plots for simulations including BGS (black bars) and the expectation under neutrality (grey bars). The right plot shows the relative difference between these two spectra across allele frequencies.

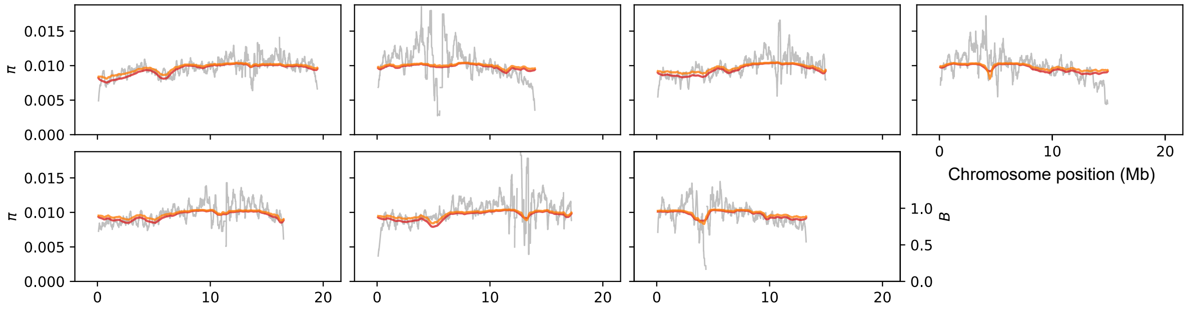

Figure S6: Levels of nucleotide diversity (π) and B in *C. grandiflora*. The grey line corresponds to observed levels of π in sliding 200 kb windows. The red line shows the prediction from the BGS-with-demography model, which includes the parameter value $\beta = 10.0$. The orange line shows the prediction when setting $\beta = 0.2$ but retaining all other parameter values.

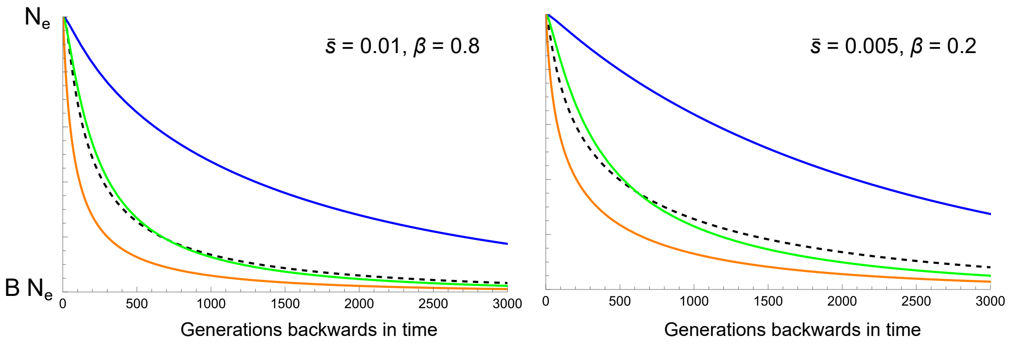

Figure S7: Approximations for the transition in coalescent N_e under BGS. The coloured lines correspond to predictions from Equation 8 (Nicolaisen and Desai 2013) when integrating across a genetic map of length 50 (orange), 0.5 (green) or 0 cM (blue). The prediction from Equation 9, which is used in the BGS-with-demography model, is shown as a black-dashed line. The two plots shows predictions for different DFE shapes, with weaker selection leading to slower transitions.

Supplementary Tables

Table S1: Maximum composite likelihood parameter estimates from fitting models of BGS to data from a simulated population with high-selfing ($\alpha = 0.9$) and a low rate of deleterious mutation ($\mu = 3.75 \times 10^{-9}$). The first row shows the parameters used in the simulation, whereas the lower rows show estimates from the BGS-with-demography and partial-selfing model and the classic-BGS model. For the classic-BGS model the estimate of N_0 (i.e. N_{max}) in brackets has been rescaled to account for partial-selfing. Note that α is estimate prior to model fitting from F_{IS} and $\bar{B}$ is a prediction from the model rather than a parameter.

	N_0	N_1	T_0	μ	$\bar{s}$	β	α	$\bar{B}$
Simulated	10,000	-	-	3.75×10^{-9}	0.0100	0.333	0.900	0.647
Demography + selfing	9138	20,022	34,069	4.24×10^{-9}	0.0051	0.232	0.902	0.653
Classic	4855 (9251)	-	-	13.42×10^{-9}	0.1044	0.689	-	0.702

Table S2: Maximum composite likelihood parameter estimates from fitting models of BGS to data from a simulated population with high-selfing ($\alpha = 0.9$) and a high rate of deleterious mutation ($\mu = 1.5 \times 10^{-8}$). See the caption of Table S1 for more details.

	N_0	N_1	T_0	μ	$\bar{s}$	β	α	$\bar{B}$
Simulated	10,000	-	-	1.50×10^{-8}	0.0100	0.333	0.900	0.222
Demography + selfing	7859	6914	473	2.21×10^{-8}	0.0095	0.136	0.903	0.307
Classic	2645 (5041)	-	-	3.15×10^{-8}	0.1133	1.444	-	0.443

Table S3: Sample information for the *Capsella orientalis* individuals analysed in this study. The *Coverage* column refers to mean read-depth, whereas the *Callable* column denotes the proportion of the *C. rubella* reference genome where genotypes can be confidently called given the coverage (≥ 10 reads but $< 3\times$ mean coverage).

Species	BioProject	BioSample	LibraryName	Run	Latitude	Longitude	Coverage	Callable
C. orientalis	PRJEB6689	SAMEA2784250	SAMEA2784250	ERR636107	51.5	81.2166667	53	0.75
C. orientalis	PRJEB6689	SAMEA2784251	SAMEA2784251	ERR636108	51.3666667	82.2	49	0.75
C. orientalis	PRJEB6689	SAMEA2784252	SAMEA2784252	ERR636109	48.5833333	88.4333333	69	0.76
C. orientalis	PRJEB6689	SAMEA2784253	SAMEA2784253	ERR636110	53.35	83.7333333	55	0.75
C. orientalis	PRJEB6689	SAMEA2784254	SAMEA2784254	ERR636111	53.35	83.7333333	55	0.75
C. orientalis	PRJEB6689	SAMEA2784255	SAMEA2784255	ERR636112	47.15	86.1166667	52	0.74
C. orientalis	PRJEB6689	SAMEA2784256	SAMEA2784256	ERR636113	47.2333333	85.7166667	57	0.75
C. orientalis	PRJEB6689	SAMEA2784257	SAMEA2784257	ERR636114	46.6166667	90.8666667	47	0.74
C. orientalis	PRJEB6689	SAMEA2784258	SAMEA2784258	ERR636115	48.5833333	88.4333333	53	0.75
C. orientalis	PRJEB6689	SAMEA2784259	SAMEA2784259	ERR636116	53.3333333	83.75	37	0.73
C. orientalis	PRJEB6689	SAMEA2784260	SAMEA2784260	ERR636117	52.2666667	76.95	53	0.75
C. orientalis	PRJEB6689	SAMEA2784261	SAMEA2784261	ERR636118	50.7833333	75.6833333	34	0.73
C. orientalis	PRJEB6689	SAMEA2784262	SAMEA2784262	ERR636119	51.1	81.9	71	0.76
C. orientalis	PRJEB6689	SAMEA2784263	SAMEA2784263	ERR636120	51.1166667	81.8	33	0.73
C. orientalis	PRJEB6689	SAMEA2784264	SAMEA2784264	ERR636121	51.1666667	81.6666667	80	0.76
C. orientalis	PRJEB6689	SAMEA2784265	SAMEA2784265	ERR636122	51.1333333	81.6	48	0.74

Table S4: Sample information for the *Capsella grandiflora* individuals analysed in this study. The *Coverage* column refers to mean read-depth, whereas the *Callable* column denotes the proportion of the *C. rubella* reference genome where genotypes can be confidently called given the coverage (≥ 10 reads but $< 3\times$ mean coverage).

Species	BioProject	BioSample	LibraryName	Run	Latitude	Longitude	Coverage	Callable
C. grandiflora	PRJNA275635	SAMN03351887	9C_dna	SRR2065335	53.071	43.998	60	0.87
C. grandiflora	PRJNA275635	SAMN03351891	8U_dna	SRR2065322	53.071	43.998	63	0.87
C. grandiflora	PRJNA275635	SAMN03351897	147Y_dna	SRR2065215	53.071	43.998	60	0.88
C. grandiflora	PRJNA275635	SAMN03351898	112H_dna	SRR2065185	53.071	43.998	60	0.88
C. grandiflora	PRJNA275635	SAMN03351899	95O_dna	SRR2065329	53.071	43.998	64	0.88
C. grandiflora	PRJNA275635	SAMN03351900	103K_dna	SRR2065174	53.071	43.998	62	0.88
C. grandiflora	PRJNA275635	SAMN03351901	63D_dna	SRR2065300	53.071	43.998	79	0.88
C. grandiflora	PRJNA275635	SAMN03351904	72G_dna	SRR2065308	53.071	43.998	59	0.87
C. grandiflora	PRJNA275635	SAMN03351906	158J_dna	SRR2065225	53.071	43.998	80	0.88
C. grandiflora	PRJNA275635	SAMN03351910	89L_dna	SRR2065321	53.071	43.998	75	0.88
C. grandiflora	PRJNA275635	SAMN03351914	177K_dna	SRR2065245	53.071	43.998	62	0.88
C. grandiflora	PRJNA275635	SAMN03351916	113A_dna	SRR2065186	53.071	43.998	64	0.88
C. grandiflora	PRJNA275635	SAMN03351921	74M_dna	SRR2065309	53.071	43.998	60	0.88
C. grandiflora	PRJNA275635	SAMN03351924	101S_dna	SRR2065172	53.071	43.998	60	0.88
C. grandiflora	PRJNA275635	SAMN03351928	181Y_dna	SRR2065248	53.071	43.998	62	0.87
C. grandiflora	PRJNA275635	SAMN03351934	154P_dna	SRR2065221	53.071	43.998	61	0.88
C. grandiflora	PRJNA275635	SAMN03351941	96O_dna	SRR2065330	53.071	43.998	59	0.88
C. grandiflora	PRJNA275635	SAMN03351955	92H_dna_1	SRR2065325	53.071	43.998	78	0.88
C. grandiflora	PRJNA275635	SAMN03351958	111C_dna_1	SRR2065183	53.071	43.998	51	0.86
C. grandiflora	PRJNA275635	SAMN03351959	93M_dna	SRR2065327	53.071	43.998	90	0.88
C. grandiflora	PRJNA275635	SAMN03351961	151D_dna	SRR2065218	53.071	43.998	69	0.88
C. grandiflora	PRJNA275635	SAMN03351963	109E_dna	SRR2065180	53.071	43.998	56	0.87
C. grandiflora	PRJNA275635	SAMN03351965	140S_dna	SRR2065209	53.071	43.998	68	0.88
C. grandiflora	PRJNA275635	SAMN03351971	24F_dna	SRR2065272	53.071	43.998	63	0.87
C. grandiflora	PRJNA275635	SAMN03351976	86I_dna	SRR2065320	53.071	43.998	64	0.88

Table S5: Sample information for the *Capsella grandiflora* individuals analysed in this study, continued from Table S4). The *Coverage* column refers to mean read-depth, whereas the *Callable* column denotes the proportion of the *C. rubella* reference genome where genotypes can be confidently called given the coverage (≥ 10 reads but $< 3\times$ mean coverage).

Species	BioProject	BioSample	LibraryName	Run	Latitude	Longitude	Coverage	Callable
C. grandiflora	PRJNA275635	SAMN03381426	31T_dna	SRR2065279	53.071	43.998	57	0.87
C. grandiflora	PRJNA275635	SAMN03381433	47K_dna	SRR2065289	53.071	43.998	66	0.88
C. grandiflora	PRJNA275635	SAMN03381437	148Z_dna	SRR2065216	53.071	43.998	61	0.87
C. grandiflora	PRJNA275635	SAMN03381438	20B_dna	SRR2065270	53.071	43.998	64	0.88
C. grandiflora	PRJNA275635	SAMN03381441	178M_dna	SRR2065246	53.071	43.998	81	0.88
C. grandiflora	PRJNA275635	SAMN03381442	141P_dna	SRR2065210	53.071	43.998	70	0.88
C. grandiflora	PRJNA275635	SAMN03381444	156R_dna	SRR2065223	53.071	43.998	70	0.88
C. grandiflora	PRJNA275635	SAMN03381446	38B_dna	SRR2065284	53.071	43.998	67	0.88
C. grandiflora	PRJNA275635	SAMN03381448	17Q_dna	SRR2065247	53.071	43.998	67	0.88
C. grandiflora	PRJNA275635	SAMN03381453	198A_dna	SRR2065262	53.071	43.998	69	0.88
C. grandiflora	PRJNA275635	SAMN03381455	35F_dna	SRR2065282	53.071	43.998	67	0.88
C. grandiflora	PRJNA275635	SAMN03381457	52A_dna	SRR2065294	53.071	43.998	59	0.88
C. grandiflora	PRJNA275635	SAMN03381462	132L_dna	SRR2065201	53.071	43.998	61	0.87
C. grandiflora	PRJNA275635	SAMN03381463	162R_dna	SRR2065229	53.071	43.998	66	0.88
C. grandiflora	PRJNA275635	SAMN03381468	28C_dna	SRR2065276	53.071	43.998	59	0.87
C. grandiflora	PRJNA275635	SAMN03775705	119M_dna	SRR2070889	53.071	43.998	65	0.88
C. grandiflora	PRJNA275635	SAMN03775713	209P_dna	SRR2070908	53.071	43.998	74	0.88
C. grandiflora	PRJNA275635	SAMN03775719	3_dna	SRR2070909	53.071	43.998	62	0.87
C. grandiflora	PRJNA275635	SAMN03775723	45_dna	SRR2070913	53.071	43.998	58	0.88
C. grandiflora	PRJNA275635	SAMN03775729	130_dna	SRR2070892	53.071	43.998	65	0.88
C. grandiflora	PRJNA275635	SAMN03775730	149_dna	SRR2070894	53.071	43.998	63	0.87
C. grandiflora	PRJNA275635	SAMN03775731	172_dna	SRR2070897	53.071	43.998	69	0.88
C. grandiflora	PRJNA275635	SAMN03775733	208_dna	SRR2070907	53.071	43.998	58	0.87
C. grandiflora	PRJNA275635	SAMN03775735	91x35_dna	SRR2070925	53.071	43.998	64	0.88
C. grandiflora	PRJNA275635	SAMN03775737	166_dna	SRR2070895	53.071	43.998	60	0.87

Table S6: Maximum composite likelihood parameter estimates from fitting the BGS-with-demography model to *C. grandiflora* data ten times. Repeated runs are sorted by descending $lnCL$.

N_0	N_1	N_2	T_0	T_1	μ	$\bar{s}$	β	$lnCL$
830,505	634,878	289,169	33,873	367,875	3.738×10^{-9}	0.003067	10.000	−5,586,958.52
831,344	635,049	289,210	33,672	367,699	3.738×10^{-9}	0.003067	10.000	−5,586,958.52
828,258	634,673	289,156	34,369	368,027	3.739×10^{-9}	0.003070	10.000	−5,586,958.52
828,426	634,919	289,205	34,221	367,686	3.738×10^{-9}	0.003068	9.937	−5,586,958.54
831,002	635,251	289,306	33,793	367,701	3.747×10^{-9}	0.003143	9.985	−5,586,958.56
832,633	635,477	289,408	33,603	367,777	3.758×10^{-9}	0.003222	9.999	−5,586,958.66
821,980	632,641	288,524	35,543	368,195	3.785×10^{-9}	0.003312	5.669	−5,586,959.17
830,080	634,986	289,259	34,050	368,013	3.749×10^{-9}	0.003212	6.221	−5,586,960.18
792,806	625,091	288,681	48,837	374,977	3.783×10^{-9}	0.003500	4.499	−5,586,962.81
684,340	278,077	2,791,517	342,698	3,101,951	3.476×10^{-9}	0.003145	10.000	−5,586,995.75

Table S7: Maximum composite likelihood parameter estimates from fitting the BGS-with-partial-selfing model to *C. orientalis* data ten times. All runs assume $\alpha = 0.981$ and are sorted by descending $lnCL$.

N_{max}	μ	$\bar{s}$	β	$lnCL$
40,329	1.540×10^{-9}	3.349×10^{-4}	0.3840	−53,220.87
40,781	1.634×10^{-9}	3.339×10^{-4}	0.3427	−53,220.88
41,457	1.934×10^{-9}	3.106×10^{-4}	0.2562	−53,220.90
39,699	1.067×10^{-9}	4.239×10^{-4}	0.8536	−53,220.93
41,963	3.437×10^{-9}	1.924×10^{-4}	0.1163	−53,220.97
42,382	3.349×10^{-9}	2.065×10^{-4}	0.1184	−53,220.98
42,098	3.667×10^{-9}	1.836×10^{-4}	0.1069	−53,220.99
42,101	3.694×10^{-9}	1.824×10^{-4}	0.1060	−53,220.99
38,748	9.532×10^{-10}	4.104×10^{-4}	1.2500	−53,221.00
104,952	3.565×10^{-9}	9.991×10^{-3}	0.1001	−53,225.77
